# Supplementary material for: Microfluidics-Based Fabrication of Cell-Laden Hydrogel Microfibers for Potential Applications in Tissue Engineering
Source: Molecules. 2019 Apr 25;24(8):1633. doi: 10.3390/molecules24081633 (PMC6515047; doi:10.3390/molecules24081633)
Supplement: Supplementary file 1 [file molecules-24-01633-s001.zip › Supplementary Materials-revised.docx]

**Supplementary Materials**


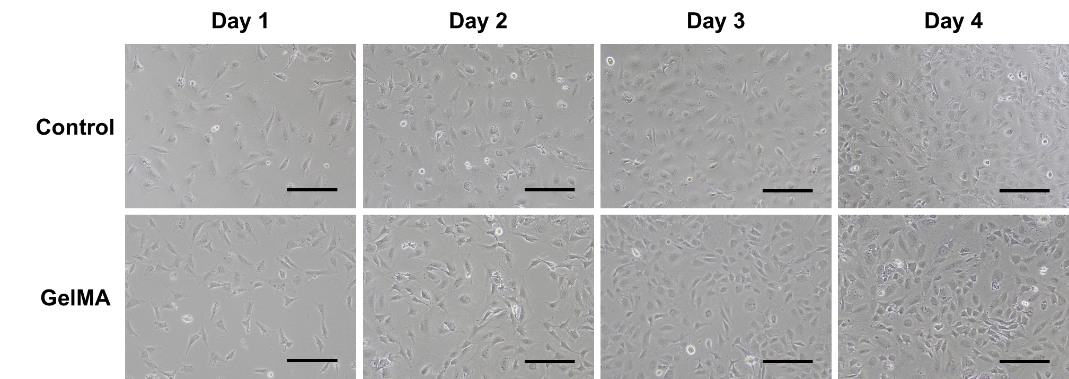


**Figure S1**. Cell culture of MC3T3-E1 cells on cell culture dish (Control) and GelMA (5%) hydrogels. Scale bars, 200 μm.


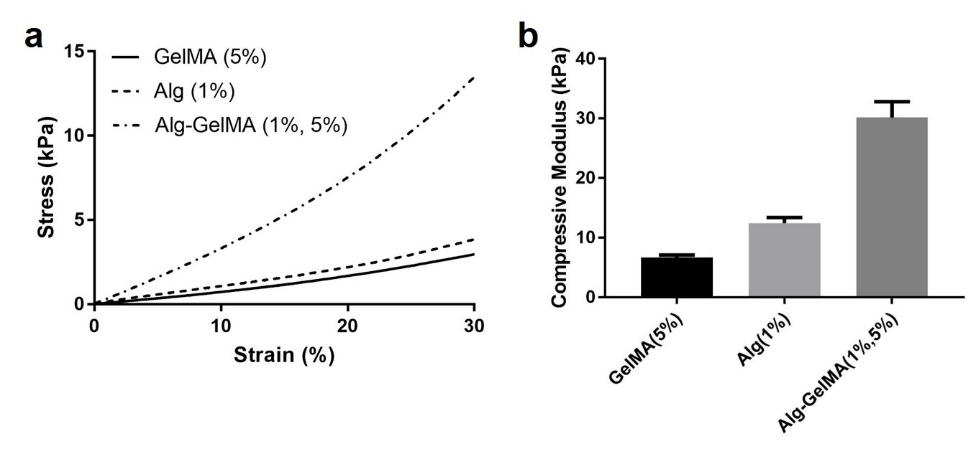


**Figure S2.** Mechanical properties of GelMA, Alg and Alg-GelMA hydrogel. Representative stress-strain curves (a) and compressive modulus (b) of GelMA (5%), Alg (1%) and Alg-GelMA (1%, 5%) hydrogel.


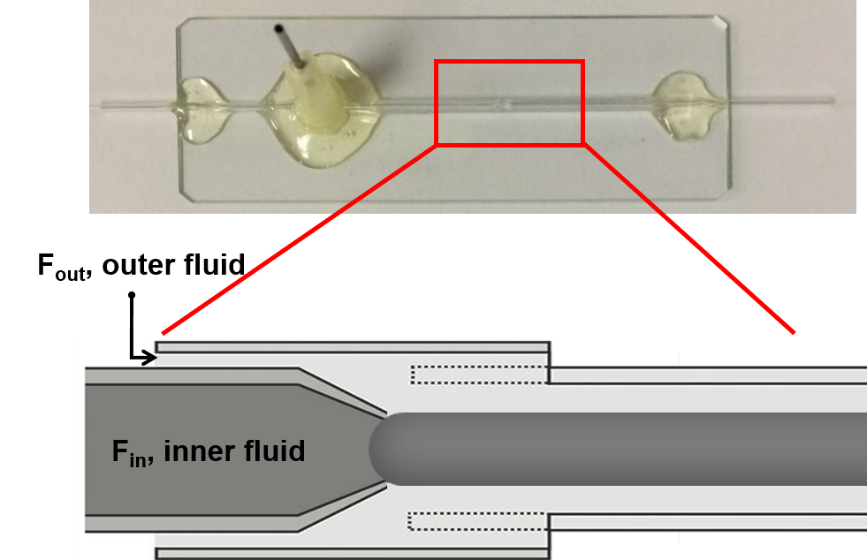


**Figure S3**. Digital photograph and schematic illustration of two-flow microfluidic devices for fabricating microfibers.

**Video S1**. Perfusion of hollow microfiber. Red fluorescent microspheres were injected into the hollow microfiber.
